# Side effects of mandibular advancement splints for the treatment of snoring and obstructive sleep apnea: a systematic review

**DOI:** 10.1590/2177-6709.23.4.045-054.oar

**Published:** 2018

**Authors:** Olivia de Freitas Mendes Martins, Cauby Maia Chaves, Rowdley Robert Pereira Rossi, Paulo Afonso Cunali, Cibele Dal-Fabbro, Lia Bittencourt

**Affiliations:** 1 Universidade Federal do Piauí, Curso de Especialização em Ortodontia (Teresina/PI, Brazil). Universidade Federal do Piauí Universidade Federal do Piauí TeresinaPI Brazil; 2 Centro Universitário UNINOVAFAPI, Curso de Especialização em Ortodontia (Teresina/PI, Brazil). Centro Universitário UNINOVAFAPI TeresinaPI Brazil; 3 Universidade Federal do Ceará, Departamento de Ortodontia (Fortaleza/CE, Brazil). Universidade Federal do Ceará Universidade Federal do Ceará Departamento de Ortodontia FortalezaCE Brazil; 4 Universidade Federal do Espírito Santo, Curso de Graduação em Odontologia (Vitória/ES, Brazil). Universidade Federal do Espírito Santo Universidade Federal do Espírito Santo VitóriaES Brazil; 5 Universidade Federal do Paraná, Departamento de Odontologia (Curitiba/PR, Brazil). Universidade Federal do Paraná Universidade Federal do Paraná Departamento de Odontologia CuritibaPR Brazil; 6 Instituto do Sono de São Paulo, Curso de Odontologia do Sono (São Paulo/SP, Brazil). Instituto do Sono de São Paulo São PauloSP Brazil; 7 Universidade Federal de São Paulo, Departamento de Psicobiologia (São Paulo/SP, Brazil). Universidade Federal de São Paulo Universidade Federal de São Paulo Departamento de Psicobiologia São PauloSP Brazil

**Keywords:** Obstructive seep apnea, Adverse effects, Mandibular advancement splints, Systematic review

## Abstract

**Introduction:**

Occlusal side effects or development of pain and/or functional impairment of the temporomandibular complex are potential reasons for poor compliance or abandonment of mandibular advancement splints treatment for snoring and obstructive sleep apnea.

**Objective:**

This study aimed at providing a comprehensive review evaluating the craniofacial side effects of oral appliance therapy for snoring and obstructive sleep apnea.

**Methods:**

An electronic search was systematically conducted in PubMed and Virtual Health Library from their inception until October 2016. Only Randomized Controlled Trials whose primary aim was to measure objectively identified side effects on craniofacial complex of a custom-made oral appliance for treating primary snoring or obstructive sleep apnea were included. Studied patients should be aged 20 or older. The risk of bias in the trials was assessed in accordance with the recommendations of The Cochrane Risk of Bias criteria.

**Results:**

A total of 62 full-text articles were assessed for eligibility. After the review process, only 6 met all the inclusion criteria. All studies were rated as having a high risk of bias. The most uniformly reported mandibular advancement splint side effects were predominantly of dental nature and included a decrease in overjet and overbite. The risk of developing pain and function impairment of the temporomandibular complex appeared limited with long-term mandibular advancement splint use.

**Conclusion:**

The limited available evidence suggests that mandibular advancement splint therapy for snoring and obstructive sleep apnea results in changes in craniofacial morphology that are predominantly dental in nature, specially on a long-term basis. Considering the chronic nature of obstructive sleep apnea and that oral appliance use might be a lifelong treatment, a thorough customized follow-up should therefore be undertaken to detect possible side effects on craniofacial complex. It is also important to provide adequate information to the patients regarding these possible changes, especially to those in whom larger occlusal changes are to be expected or in whom they are unfavorable. Long-term assessments of adverse effects of oral appliance therapy, with larger study samples and recruitment of homogenous patient population are still required.

## RESUMO

**Introdução:**

efeitos colaterais oclusais e o desenvolvimento de dor e/ou disfunção do complexo temporomandibular podem levar à baixa adesão ou ao abandono do tratamento do ronco e da apneia obstrutiva do sono com aparelhos de avanço mandibular.

**Objetivo:**

fornecer uma revisão abrangente da literatura sobre os efeitos colaterais craniofaciais do tratamento do ronco e da apneia obstrutiva do sono com aparelhos de avanço mandibular.

**Métodos:**

foram realizadas buscas eletrônicas sistematicamente no PubMed e na Biblioteca Virtual em Saúde até outubro de 2016. Foram incluídos apenas Ensaios Controlados Randomizados, com o objetivo primário de mensurar objetivamente os efeitos colaterais no complexo craniofacial associados ao uso de aparelhos de avanço mandibular no tratamento do ronco e da apneia obstrutiva do sono. Os pacientes estudados deveriam ter 20 anos de idade ou mais. A avaliação do risco de viés dos trabalhos selecionados seguiu as recomendações do The Cochrane Risk of Bias.

**Resultados:**

no total, 62 artigos completos foram avaliados em relação à elegibilidade. Após o processo de revisão, apenas 6 atenderam aos critérios de inclusão. Todos os estudos foram julgados como tendo alto risco de viés. Os efeitos colaterais mais frequentemente encontrados foram de natureza dentária e incluíram uma diminuição do *overjet* e do *overbite*. O risco de desenvolvimento de dor ou disfunção do complexo temporomandibular pareceu limitado na avaliação de longo prazo do uso do aparelho de avanço mandibular.

**Conclusão:**

as evidências disponíveis são limitadas e sugerem que o tratamento do ronco e da apneia obstrutiva do sono com aparelhos de avanço mandibular resulta em alterações craniofaciais predominantemente dentárias, especialmente nas avaliações de longo prazo. Considerando-se que a apneia obstrutiva do sono é crônica e que os aparelho intrabucais se constituem em uma forma de tratamento contínuo e por tempo indefinido, é necessário um acompanhamento individualizado para monitorar possíveis efeitos colaterais no complexo craniofacial. Também é importante informar aos pacientes sobre esses possíveis efeitos, especialmente àqueles nos quais são esperadas maiores alterações oclusais ou nos quais elas sejam desfavoráveis. Ainda são necessárias avaliações de longo prazo dos efeitos colaterais do tratamento com aparelhos intrabucais, com amostras maiores e mais homogêneas.

## INTRODUCTION

Over the past decade, mandibular advancement splints (MAS) have been enthusiastically studied and considered as a simple, silent, bed partner-friendly, less invasive, tolerable, and efficacious choice for snoring and mild-to-moderate obstructive sleep apnea (OSA).^1^ The current literature increasingly supports MAS as an effective alternative to Continuous Positive Airway Pressure (CPAP) for patients with mild or moderate OSA.^2^^,^^3^ In a 2-year randomized trial of 103 patients, Doff et al^2^ compared subjective and objective treatment outcome of oral appliance (OA) therapy and CPAP in patients with OSA. They found that there was no statistical difference between treatments neither in the proportion of patients obtaining successful treatment (56% vs 60% in non-severe, and 50% vs 75% in severe for MAS and CPAP, respectively), nor in terms of subjective parameters for improvements in sleepiness, functional outcomes, and health perceptions (Epworth Sleepiness Scale, Functional Outcomes of Sleep Questionnaire, Medical Outcomes Study 36-item Short Form Health Survey [SF-36]). However, CPAP was more effective in lowering the apnea / hypopnea index and showed higher oxyhemoglobin saturation levels compared to OA therapy. It could be hypothesized that the suboptimal efficacy with MAS therapy is counteracted by the greater compliance with OA relative to CPAP, resulting in similar effectiveness of both treatments.^2^

Based on their mechanism of action, OA for OSA can be grouped into two categories: tongue retaining appliances and mandibular advancement splints (MAS). Tongue retaining appliances are currently seldom used, mainly due to patient intolerance, being almost completely replaced by MAS.^3^^,^^4^ The quantity and quality of scientific literature supporting their use is far greater than for the other types of devices.^5^

MAS are anchored to dentition and hold the mandible in a forward, vertically open position, pulling forward the tongue base and stretching pharyngeal soft tissues.^1^^,^^4^^,^^6^ Therefore, as these devices hold the mandible in an advanced and vertically opened position, they generate a continuously load to teeth and surrounding tissues by means of the traction forces exerted by the masticatory and mylohyoid muscles and soft tissue, which pull the mandible posteriorly into its habitual position,^2^^,^^4^^,^^7^ particularly during swallowing overnight. One force is labially directed to the mandibular incisors and the other is palatally directed to the maxillary incisors^2^^,^^8^. It has been hypothesized that this may change the inclination and position of teeth, affect the position of the mandible and increase the loading to the craniomandibular complex.^7^

In the initial period of MAS use, patients usually report tenderness of the teeth and jaws, gum irritation, excessive salivation or dry mouth^8^. Nevertheless, these complaints are usually mild, acceptable and disappear with continued use, or they are easily resolved when addressed by the dentist^5^. On a long-term basis, MAS therapy may result in objective adverse effects such as tooth movement, skeletal changes, and occlusal alteration.^9^

Temporomandibular disorders (TMD) have been associated with the use of MAS. Doff et al^10^ assessed in patients with OSA the occurrence of TMD due to long-term use (2-year follow-up) of an OA compared to CPAP therapy. They found that OA therapy resulted in more pain-related TMD in the initial period of use compared with CPAP therapy, although generally it was not serious and it had a transient nature (tended to decrease afterwards). Moreover, mandibular function was not impaired in OSA patients using an OA or CPAP therapy. Therefore, authors suggest that the possible development of TMD or temporary pain of the temporomandibular complex is not a contra-indication for OA therapy in OSA patients. 

Although the main reason for patients to stop wearing the OA is its ineffectiveness,^3^ potential reasons for poor compliance or discontinuation of MAS treatment are occlusal side effects or development pain and/or functional impairment of the temporomandibular complex. Furthermore, it is important a better understanding of the precise craniofacial effects of this therapy considering the chronic nature of OSA and that MAS use might be a lifelong treatment, aimed at helping dentists who treat sleep-disorder breathing to better understand the possible skeletal and dental/occlusal changes, as well the developing of pain and function impairment of the temporomandibular complex related to MAS therapy. 

A 2004 systematic review^5^ investigated the safety of OA therapy in patients with OSA. Thirteen studies were included for methodological appraisal, being one controlled clinical trial and twelve case series. Authors concluded that MAS therapy may result in adverse (although generally not serious) effects on the craniomandibular and craniofacial complex, which generally involve changes in dental occlusion. They also pointed out that controlled studies addressing co-morbidity of OA therapy were needed. Since this publication, recent articles have been published regarding the side effects of MAS as a treatment modality for OSA.^2^^,^^4^^,^^7^^,^^9^^,^^10^ Therefore, the aim of this study was to systematically review the available literature concerning the safety of MAS therapy for snoring and OSA.

## METHODS

This systematic review followed The Preferred Reporting Items for Systematic reviews and Meta-Analyses for Protocols 2015 (PRISMA-P 2015).^11^

### Protocol and registration

Protocol and registration were not performed.

### Eligibility criteria

The Population, Intervention, Comparison, Outcome and Study design (PICO) question format was used to formulate a clinical question and to elaborate inclusion criteria.

In adults (aged 20 or older) with OSA or snoring treated with MAS, what are the side effects on craniofacial complex measured objectively by clinical examination, cephalometric analyses and dental cast measurements?

» Population/patient: Adults (aged 20 or older) with OSA or snoring.
» Intervention: Treatment with a MAS.
» Comparison: Treatment *versus* control (CPAP, placebo or inactive appliance, uvulopalatopharyngoplasty) or before and after treatment.
» Outcome: Side effects on craniofacial complex measured objectively by clinical examination, cephalometric analyses and dental cast measurements.
» Study design: randomized clinical trials (RCTs).

### Selection criteria

The inclusion criteria were: (1) studies whose primary aim was to measure objectively identified side effects on craniofacial complex of MAS for treating OSA; (2) studied patients diagnosed with OSA or primary snoring; (3) studied patients aged 20 or older; (4) intervention group treated with a custom-made MAS; (5) RCTs. The exclusion criteria were: (1) studies regarding only patient-perceived co-morbidity of this therapeutic modality (trials with nonclinical outcomes); (2) intervention group treated utilizing both mandibular protrusion and tongue retention. The search strategy was not limited to RCTs so that the reference lists of all articles obtained could be manually searched.

### Information sources and search strategy

**Figure 1:**
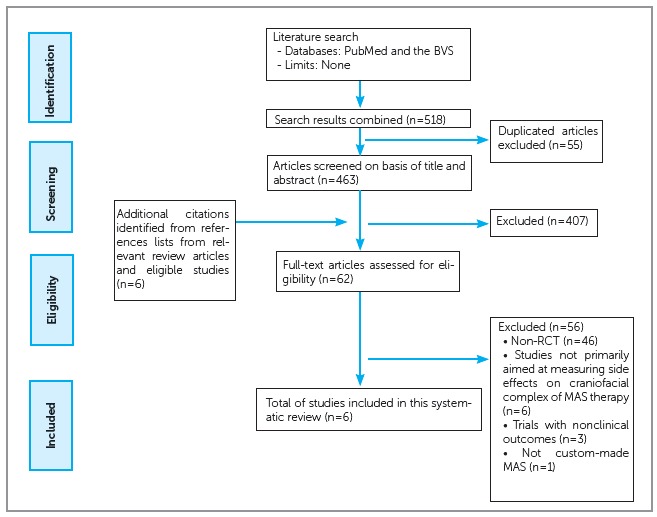
Flow diagram of selection process.

Search was performed on PubMed and the BVS (Virtual Health Library), that includes the databases LILACS, MEDLINE and Cochrane Library, from their inception until October 2016 (Fig 1). The search strategy for PubMed was conducted using medical subject headings (MeSH) and subheadings. Similar search terms were adopted for the other database. In addition, reference lists from relevant review articles and eligible studies was checked to identify any additional citations that might have been missed. No limits were applied to any of the search strategy.

### Study selection

In the first step of the screening process, duplicated publications were excluded and two reviewers independently screened titles and abstracts, to identify full articles whose main purpose was to identify side effects on craniofacial complex of mandibular advancement for treating OSA. Any disagreements were resolved by a third reviewer. The same two authors evaluated the full text articles independently by applying the remaining inclusion criteria listed above. No limits were applied for language and foreign papers (not English) were translated.

### Data items

For each selected trial, the following data were collected (Table 1): (1) first author and publication year; (2) methods (clinical examination, cephalometric analyses and dental cast measurements); (3) MAS type and amount of protrusion; (4) type and number of subjects; (5) control; (6) follow-up period; (7) MAS use (including nights/week and hours/night and study duration); and (8) side effects summary.

**Table 1:** Summary of descriptive characteristics of included studies

Author (year)	Method	MAS		Subjects		Control	Follow-up period	MAS Use	Side effects summary
		Type	Protrusion	Type	n				
Doff et al.^16^ (2010)	Cephalometry	Thornton Adjustable Positioner (TAP®)	79 ± 20% of maximum protrusion	OSA	31	CPAP	2.3 ± 0.2 year	> 6 nights/week	Overbite and overjet decreased; retroclination of the upper incisors and proclination of the lower incisors; lower and total anterior facial height increased significantly; no changes in skeletal variables were found
Doff et al.^10^ (2012)	Clinical measurements (RDC/TMD) and questionnaire	Thornton Adjustable Positioner (TAP®)	76 ± 25% of maximum protrusion	OSA	51	CPAP	2 months, 1 year and 2 years	6.7 ± 0.6 nights/week	OA therapy results in more pain-related TMDs in the initial period of use, compared with CPAP therapy
Doff et al.^17^ (2013)	Dental plaster study models	Thornton Adjustable Positioner (TAP®)	79 ± 19% of maximum protrusion	OSA	51	CPAP	2.3 ± 0.2 year	6.9 ± 0.4 nights/week	Decreased overjet/overbite, anteroposterior change in the occlusion, decreased number of occlusal contact points in the posterior region
Ringqvist et al.^14^ (2003)	Cephalometry	Non- adjustable	50% of maximum protrusion	OSA	27	Uvulopala-topharyn-goplasty	4.1 year (4.0 - 4.2)	6.1 nights/week	Changes in vertical positions of the maxillary incisors and the mandibular incisors, posterior rotation of the mandible; overjet and overbite did not change significantly
Robertson et al.^13^ (2003)	Cephalometry	Non- adjustable	75% of maximum protrusion	OSA/ snoring	100	Pre-MAS therapy	6, 12, 18, 24 and 30 months	> 5-6 hours/nights 7 nights/week	Changes in face height, the position of the mandible, overjet, and overbite occurred as early as 6 months. Over-eruption of the maxillary first premolars and mandibular first molars and proclination of the mandibular incisors were not evident for at least 2 years.
Tegelberg et al.^15^ (1999)	Clinical examination (Helkimo; Eichner index of occlusal support zones)	Non- adjustable	50% of maximum protrusion	OSA	37	Uvulopala-topharyn-goplasty	1 year	6 nights/week	Few adverse events in the stomatognathic system or other complications

### Data collection process

Data was extracted by two reviewers and then it was combined and compared for accuracy. Any disagreements were resolved by a third reviewer.

### Risk of bias in individual studies

Two authors independently evaluated the risk of bias in the included studies following the recommendations of The Cochrane Risk of Bias criteria.^12^ Disagreements were resolved by a third reviewer.

### Data synthesis

If the collected data was found to be adequate, a meta-analysis was considered.

## RESULTS

### Study selection

A flow diagram illustrating the number of citations identified, screened, and included in this review is outlined in Figure 1. A total of 518 articles were selected using the search strategy specified before. Of these, 55 were duplicated and were excluded. Six additional citations were identified from the references lists of relevant articles. Were discarded 407 articles after reviewing the titles and/or abstracts, remaining 62 articles of possible interest. Six studies finally met all the inclusion criteria and were included in this review. 

### Study characteristics

A summary of study characteristics of included trials is found in Table 1. Included articles were all in English language and most of them were conducted after the year 2000. 

In most studies^13^^,^^14^^,^^17^ only patients with mild to moderate OSA or asymptomatic snores were included and it was used a non-adjustable OA that fixed the mandible in a predefined position at 50^14^^,^^17^ or 75%^13^ of the maximum mandibular protrusion. The studies conducted by Doff et al^10^^,^^16^^,^^17^ compared possible adverse effects of a titratable OA with a CPAP control group in patients with mild to severe OSA. They used an adjustable OA and the mean mandibular protrusion during the follow-up period was 79 ± 20% of the maximal mandibular protrusion. They also studied the relationship between the occurrence of these effects and the degree of mandibular protrusion during OA therapy.

Two of the included articles^10^^,^^15^ studied possible side effects of MAS therapy on function and morphology of temporomandibular joint (TMJ) and masticatory muscles by clinical examination, including measurements of mandibular mobility, palpation of TMJ joints and masticatory muscles, and registrations of pain on mobility. In one of these two studies, clinical examination was quantified by the Clinical Dysfunction Score (CDS, Helkimo 1974),^15^ whereas the other^10^ used the Research Diagnostic Criteria for Temporomandibular Disorders (RDC/TMD), which evaluates TMD in a standardized manner.^10^ In three studies, dental/occlusal and skeletal adverse effects of MAS therapy were assessed by means of cephalometry,^13^^,^^14^^,^^16^ and in one study it was done by means of plaster cast measurements.^17^

The duration of the included studies in this review was variable and ranged from 6 months^13^ to a mean follow-up of 4 years^14^. Two study tried to determine the time course of adverse effects. Robertson et al^13^ took the review cephalogram at six month intervals (6, 12, 18, 24 or 30 months after placement of OA) to establish whether adverse effects were progressive with continuing treatment. Doff et al^10^ recorded TMD, pain intensity and disability and mandibular function impairment, at baseline, after 2 months, 1 year and 2 years of therapy.

### Risk of bias within studies

Risk of bias assessment is shown in Table 2. All included studies were found to have high risk of bias potential. None of the trials described the method of randomization neither described allocation concealment, and they were all found to have high risk of bias.

**Tabele 2:** Risk of bias assessment.

Characteristic	Study		
	Doff et al,^16^ 2010	Doff et al,^10^ 2012	Doff et al,^17^ 2013
Sequence generation (selection bias)	Unclear	Unclear	Unclear
Allocation concealment (selection bias)	Unclear	Unclear	Unclear
Blinding of participants and personnel (performance bias)	High: performance bias due to knowledge of the allocated interventions by participants and personnel during the study	High: performance bias due to knowledge of the allocated interventions by participants and personnel during the study	High: performance bias due to knowledge of the allocated interventions by participants and personnel during the study
Blinding of outcome assessment (detection bias)	Low: one blinded observer performed all tracings	Unclear: unclear if outcome assessor was blinded	Low: one blinded observer performed twice all measurements
Incomplete outcome data (attrition bias)	Low: reasons for withdrawals were both reported and balanced across groups	Low: reasons for withdrawals were both reported and balanced across groups	Low: reasons for withdrawals were both reported and balanced across groups
Selective outcome reporting (reporting bias)	Low: pre-specified outcomes were reported	Low: pre-specified outcomes were reported	Low: pre-specified outcomes were reported
Other sources of bias	High: inter- and intraobserver reliability measurements were not carried out	Unclear	Unclear
Overall risk of bias	High	High	High
Characteristic	Study		
	Ringqvist et al,^14^ 2003	Robertson et al,^13^ 2003	Tegelberg et al,^15^ 1999
Sequence generation (selection bias)	Unclear	Unclear	Unclear
Allocation concealment (selection bias)	Unclear	Unclear	Unclear
Blinding of participants and personnel (performance bias)	High: performance bias due to knowledge of the allocated interventions by participants and personnel during the study	High: performance bias due to knowledge of the allocated interventions by participants and personnel during the study	High: performance bias due to knowledge of the allocated interventions by participants and personnel during the study
Blinding of outcome assessment (detection bias)	Unclear: unclear if outcome assessor was blinded	Unclear: unclear if outcome assessor was blinded	Unclear: unclear if outcome assessor was blinded
Incomplete outcome data (attrition bias)	High: large number of patients lost to follow up (33% in MAS group)	Low: no missing outcome data	Low: reasons for withdrawals are both reported and balanced across groups
Selective outcome reporting (reporting bias)	Low: pre-specified outcomes were reported	Low: pre-specified outcomes were reported	High
Other sources of bias	High: in the experimental (MAS) and control (UPPP) groups, some patients (10% and 27%) received both treatments	High: no control group	Unclear
Overall risk of bias	High	High	High

### Synthesis of results

Since the available collected information was found not to be adequate (high methodological heterogeneity among the studies that could be compared), a meta-analysis was not possible. The reported results are descriptive in nature.

Doff et al^10^ studied variations in the occurrence of TMDs and the risk of developing pain and function impairment of the temporomandibular complex in OSA patients treated with either a MAS or CPAP. They found that in the initial period after initiating OA or CPAP therapy (2 to 3 months), the occurrence of pain-related TMDs increases, being substantially higher (24%) in the OA group than in the CPAP group (6%). These findings correspond with the results of Tegelberg et al,^15^ which show that in the short-term MAS is likely to cause pain-related signs and symptoms of TMD, but these symptoms are characterized as mild and transient. 

At the 12-month follow-up, Tegelberg et al^15^ observed that the mandibular movements, such as mouth opening, laterotrusion, and protrusion, did not change. The CDS was low before treatment and remained low during all observation period. However, authors emphasized that in the terminal phase of the study two patients developed a severe CDS and needed supplementary treatment with non-steroid anti-inflammatory drugs. This indicates that there may occur complaints regarding the masticatory muscles and the TMJs also when the mandible is moderately advanced.

Robertson et al^13^ randomly assigned one hundred adults with OSA and/or asymptomatic snoring to a group and reviewed 6, 12, 18, 24 or 30 months after placement of a non-adjustable MAS. There were 20 subjects in each group. Craniofacial changes were measured on lateral cephalometric radiographs taken at the initial and review appointments. The differences between each subject’s review and initial film were used to determine the skeletal, dental and occlusal changes. There was considerable variation both within and between groups when the changes over time were examined. Reductions in overbite (OB) and overjet (OJ), 0.61 and 0.87 mm, respectively, were evident at 6 months.

Studies included in this review^10^^,^^15^ has shown that MAS use appear to be not detrimental to TMJ health and function in OSA patients on a long-term basis. Doff et al^10^ assessed the occurrence of TMD as result of long-term use of a MAS compared to CPAP in OSA patients and found that the occurrence of TMD-related pain increases in the initial period of OA therapy but tends to return to baseline values during a 2-year follow-up. They also showed that OA therapy does not cause pain-induced limitations of the temporomandibular complex and no differences were found in mandibular function impairment during the 2-year follow-up between OA and CPAP therapy.

Nearly all studies included in this review that investigated dental changes have found a significant change in the relationship between maxillary and mandibular incisors, with decreased OB and OJ^13^^,^^16^^,^^17^. Reductions in OB and OJ ranged from 1.0 to 1.2 mm and 1.06 to 1.7 mm, respectively, after long-term appliance use. It is generally hypothesized that these changes can be attributed to a labially directed force to the mandibular incisors and a palatally directed force to the maxillary incisors during OA therapy while the mandible attempts to return to a more dorsal position^16^. On the other hand, Ringqvist et al^14^ did not observe significant changes in these measurements after 4 years of OA therapy.

Doff et al^17^ observed a more mesial molar and canine relationship after long-term use of MAS. The consequence is a Class III tendency, with a shift in molar and cuspid occlusion from Class I to Class III or a shift from Class II to Class I or III.^17^

A significant retroclination of the maxillary incisors and proclination of the mandibular incisors were observed in the majority of studies that investigated dental changes.^13^^,^^16^^,^^17^ Doff et al^16^ found a retroclination of -2.0 ± 2.8^o^ for the upper incisors and a proclination of 3.7 ± 5.4^o^ for the lower incisors.

The bite tends to open in the (pre)molar region, resulting in a decrease in the number of occlusal contact points in the (pre)molar region.^17^ A tendency towards the development of a (bi)lateral crossbite in the (pre) molar region has been found after long-term OA use.^17^

Downward rotation of the mandible and increasing in lower facial height has been observed following long-term MAS therapy.^13^^,^^14^^,^^15^

## DISCUSSION

In this systematic review, the available evidence about the craniofacial side effects of MAS therapy for snoring and OSA was investigated. Since quantity and quality of scientific literature supporting MAS use is far greater than for other types of devices^5^, this review was limited to the side effects of this kind of appliances for the treatment of OSA. 

Following selection process, only a limited number of articles was found and most of them with methodological restrictions. It is important to note that non-adjustable appliances that fix the mandible in a predefined position at 50-75% of the maximum mandibular protrusion were used in three studies,^13^^,^^14^^,^^15^ and their results may not be fully comparable with those in which totally adjustable appliances were used.^10^^,^^16^^,^^17^ Furthermore, those trials^13^^,^^14^^,^^15^ included only patients with non-apneic snoring or mild to moderate OSA, while the others^10^^,^^16^^,^^17^ included moderate to severe OSA, and protrusive positions of the mandible over 75% of the patient’s maximum mandibular protrusion were applied in some patients. Severe OSA patients may need more pronounced protrusive positions of the mandible in order to experience sufficient benefit from MAS therapy.^16^

For didactic purposes, side effects of MAS treatment were divided into two parts: (1) short/medium-term effects of MAS treatment (follow-up ≤ 1 year); and (2) long-term side effects of MAS treatment (follow-up > 1 year). Within each category, side effects were divided into three sections: (1) TMDs; (2) dental/occlusal changes; and (3) skeletal changes. 

### Short/medium term side effects

Studies with a follow-up up to 1-year (≤ 1 year) were included in this section. 

#### Temporomandibular disorders

Studies included in this review have shown that in the short-term MAS are likely to cause pain-related signs and symptoms of TMD, but these symptoms are characterized as mild and transient.^10^^,^^15^ Two possible explanation for this finding have been presented: (1) the strain in the muscles of the temporomandibular complex or the capsular ligament of the TMJ when protruding the mandible during sleep; and (2) an increase in occlusal vertical dimension while wearing OA.^10^

#### Dental/occlusal changes 

Only one study included in this review addressed dental/occlusal changes on a short-term basis (follow-up ≤ 1 year). Robertson et al^13^ observed that reductions in OB and OJ (0.61 and 0.87mm, respectively) and reductions in maxillary arch length were evident as early as after 6 months of treatment. These changes were attributed to the appliance acting directly on the incisors, with significant retroclination of the maxillary incisors and proclination of the mandibular incisors. Increased pressure from the lips due to the altered mandibular posture might also play a part.^13^

#### Skeletal changes

Robertson et al^13^ observed that skeletal changes occurred soon after the onset of treatment (6 months). Small but statistically significant increases in face height were accompanied by a significant downward position of the mandible. At 12-month follow-up, only downward displacement of the mandibular symphysis was found. However, the authors point out that, based in the findings from this study, the so-called skeletal and mandibular positional changes can be mainly attributed to appliance-induced dental changes.

### Long-term side effects

This section included side effects observed in studies with a follow up of more than 1 year (> 1 year). They were also grouped into the same sections as the short-term/medium effects (TMD, dental/occlusal changes, and skeletal changes). 

#### Temporomandibular disorders 

Studies included in this review^10^^,^^15^ have shown that MAS use seems to be not detrimental to TMJ health and function in OSA patients on a long-term basis. Although in the short-term MAS are likely to cause pain-related signs and symptoms of TMD, all pain-related TMD had decreased after 1 year^10^^,^^15^ and 2 year^10^ of using MAS. Doff et al^10^ hypothesized that the temporomandibular complex has adaptive capacities to the unnatural protrusive position during sleep while wearing an OA, and thus the device could have a therapeutic effect in patients with TMD. 

#### Dental/occlusal changes

Most studies included in this review have found a significant change in the relationship between maxillary and mandibular incisors, with reductions in OB and OJ.^13^^,^^14^^,^^16^^,^^17^ It is generally hypothesized that these changes can be attributed to a labially directed force to the mandibular incisors and a palatally directed force to the maxillary incisors during OA therapy while the mandible attempts to return to a more dorsal position.^17^ On the other hand, Ringqvist et al^14^ did not observe significant changes at anterior teeth after 4 years of OA therapy. Differences in appliance design and amount of protrusion would explain these findings. Ringqvist et al^14^ used an OA in which both frontal parts of upper and lower dental arches were not covered by acrylate, possibly resulting in less forces applied to the upper and lower incisors. Another explanation could be the degree of mandibular protrusion of 50% while wearing the OA^14^*versus* protrusive positions of the mandible over 75% of the patient’s maximum mandibular protrusion applied in some patients in the former studies.^16^^,^^17^

Robertson et al^13^ observed a significant positive correlation between the amount of anterior opening by the MAS and changes in OB at 24 and 30 months follow-up. Therefore, it was postulated that the changes in OB could be lessened by keeping the bite opening to a minimum. Furthermore, by using linear regression analysis, Doff et al^16^ observed an association between the mean amount of mandibular protrusion during the follow-up period (2.3 ± 0.2 years) and the decrease in OB. This result confirms the finding of a previous cephalometric study conducted in the same patient population.^10^ Findings of these two trials suggest that it is important to use a sagittally adjustable OA (stepwise advancement) that allow for individual titration. That approach lowers the risk of mandibular advancement beyond the optimal degree of protrusion.

A tendency towards the development of a (bi)lateral crossbite in the (pre)molar region has been found after long-term OA use.^17^ It could be explained by the fact that, with a mesial shift in occlusion, the broader part of the mandibular dental arch will occlude with the narrower part of the maxillary dental arch.^17^

The bite tends to open in the (pre)molar region, resulting in a significant decrease in the number of occlusal contact areas.^17^ It occurs probably because of the incisal guidance phenomenon (decrease in OJ and OB). However, it has been suggested that the open bite tendency tend to stabilize over time because of the development of a new occlusal equilibrium.^17^

It is important to emphasize that not all occlusal changes resulting from long-term OA therapy should be interpreted as unfavourable.^9^ In other words, some effects can lead to beneficial orthodontic changes in specific cases. For example, decreasing in overjet effects and the tendency towards a mesiocclusion (more mesial molar and canine) are favorable for Class II division 1 patients. Therefore, effects of OAs may be considered on an individual basis. Additionally, patient perceptions to occlusal changes do not correlate with objective measurements.^8^

#### Skeletal changes

Downward rotation of the mandible and increases in lower facial height have been observed following long-term MAS therapy.^13^^,^^14^^,^^16^ Ringqvist et al^14^ hypothesized that it would have been an effect of the anchorage of the MAS at the molars (well-fitting Adams clasps), with extrusion of the molars as patients attempted to open their mouth during sleep. Doff et al^16^ also believe that these skeletal changes were predominantly the result of dental changes, such as proclination of the mandibular incisors, retroclination of the maxillary incisors, and molar extrusion. 

Since treatment of OSA with MAS is probably a long-lasting process, a thorough customized follow-up should therefore be undertaken to detect possible side effects on craniofacial complex.

## LIMITATIONS 

Some methodological limitations of this review should be mentioned. Electronic search was performed in only two databases and grey literature was not searched. In an attempt to avoid as much selection bias as possible, the search strategy was not limited to RCTs, reference lists from relevant review articles and eligible studies were manually searched, and no limits were applied to any of the search strategy. Also, due to methodological and clinical heterogeneity among the studies that could be compared, the synthesis of results was considered unreliable.

## CONCLUSIONS

The limited available evidence suggests that MAS therapy for snoring and OSA results in changes in craniofacial morphology that are predominantly dental in nature, particularly on a long-term basis. It is important to provide adequate information to the patients regarding these possible changes, especially to those in whom larger occlusal changes are to be expected or in whom they are unfavorable. Long-term assessments of adverse effects of MAS therapy with larger study samples and recruitment of homogenous patient population are still required.
